# Performance evaluation of multi-junction solar cells by spatially resolved electroluminescence microscopy

**DOI:** 10.1186/s11671-014-0719-9

**Published:** 2015-02-05

**Authors:** Lijing Kong, Zhiming Wu, Shanshan Chen, Yiyan Cao, Yong Zhang, Heng Li, Junyong Kang

**Affiliations:** Department of Physics, Fujian Key Laboratory of Semiconductor Materials and Applications, Xiamen University, Xiamen, 361005 People’s Republic of China; Xiamen Changelight Co., Ltd, Xiamen, 361101 People’s Republic of China

**Keywords:** Multi-junction solar cells, Electroluminescence imaging, Quantum efficiency, Current matching

## Abstract

An electroluminescence microscopy combined with a spectroscopy was developed to visually analyze multi-junction solar cells. Triple-junction solar cells with different conversion efficiencies were characterized by using this system. The results showed that the mechanical damages and material defects in solar cells can be clearly distinguished, indicating a high-resolution imaging. The external quantum efficiency (EQE) measurements demonstrated that different types of defects or damages impacted cell performance in various degrees and the electric leakage mostly degraded the EQE. Meanwhile, we analyzed the relationship between electroluminescence intensity and short-circuit current density *J*_SC_. The results indicated that the gray value of the electroluminescence image corresponding to the intensity was almost proportional to *J*_SC_. This technology provides a potential way to evaluate the current matching status of multi-junction solar cells.

## Background

Multi-junction (MJ) solar cells have attracted broad interests, owing to their high conversion efficiency and wide future applications [1-8]. Generally, MJ solar cells consist of multiple thin semiconductor films, and the semiconductor in each junction has a characteristic bandgap, which only absorbs sunlight with the energy larger than its bandgap. The combination of several different semiconductor layers enables the solar cell to absorb sunlight efficiently and consequently improves the conversion efficiency. Up to now, the most popular MJ solar cells are based on III-V semiconductors (e.g., GaInP/GaInAs) epitaxially grown on single crystalline Ge substrate [5,6]. As is known to all, their efficiencies significantly depend on the crystal quality, electrode structure, and current matching status. However, due to their complex structures and manufacturing processes, the characterization of these devices as well as current matching remains extremely challenging, especially for an experimental access to the information of individual subcells in an MJ solar cell. Therefore, a fast, efficient and nondestructive detection technology used to derive the individual subcell electrical characteristics has a significant bright prospect.

In this work, we propose a method to characterize and evaluate the properties of each subcell in MJ solar cells by combining electroluminescence (EL) microscopy and spectroscopy. Four GaInP/GaInAs/Ge solar cells with different conversion efficiencies were systematically studied. The intrinsic defects and damages during the fabrication process could be conveniently recognized by comparison of the EL images of each junction. The external quantum efficiencies (EQEs) of these samples were measured, and the influence of defects on EQE was discussed as well. In addition, the theoretical relation between EL intensity and quantum efficiency was also deduced. It is believed that the EL imaging technique provides a pertinent and nondestructive means to characterize MJ solar cells.

## Methods

### Experimental equipment

**Figure 1:**
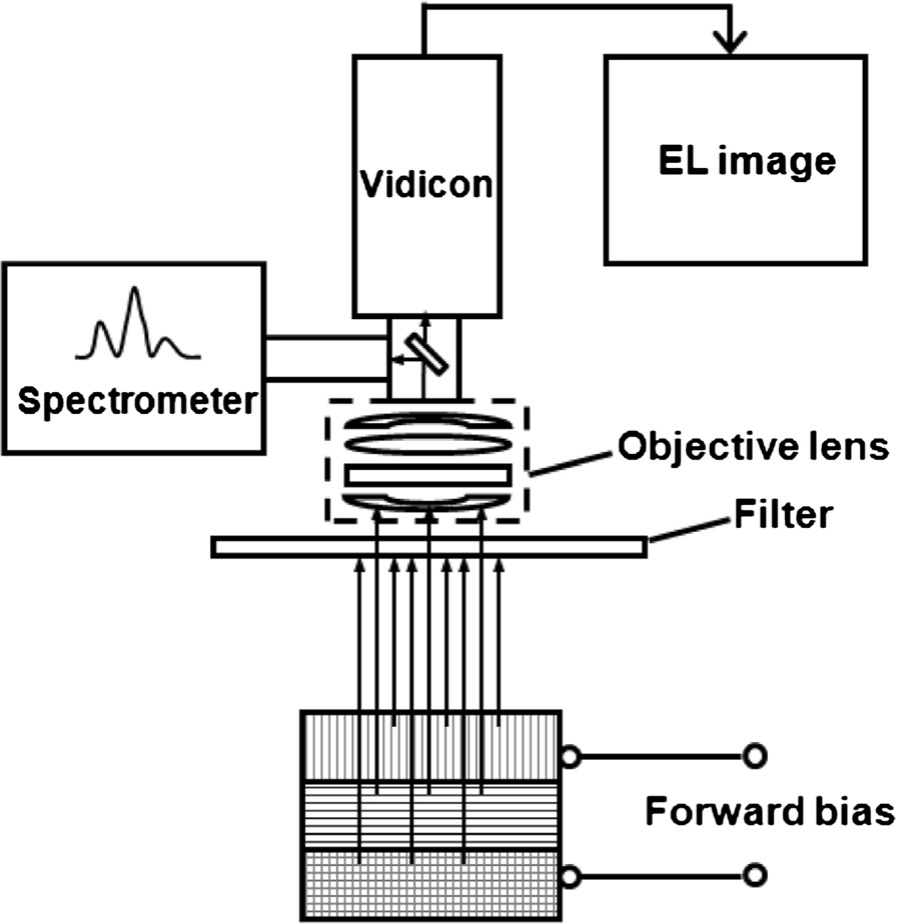
**Schematic illustration of the EL setup.**

Figure 1 shows the schematic of the EL measurement setup. The triple-junction (TJ) solar cell biased at an appropriate forward voltage emits light within three different wavelength regions, which is collected by a vidicon. One visible vidicon (inspection range: 400 to 800 nm) and an infrared (IR) vidicon (inspection range: 400 to 1,800 nm) together with several IR filters (750 to 2,750 nm) are used to specify the desirable wavelength range. Selected lenses could be fitted with spacer rings for better adjustment of the image, so that the observable range of sample size is available from millimeter to meter. When a microscope system is equipped as a substitute for the lenses, the spatial resolution even could be further improved to micrometer level. An EL spectrometer with a wavelength range from 200 to 1,100 nm is also attached to the EL image system to record the EL spectrum. The EQE was measured on a broadband spectroscopy system composed of a grating monochromator (Spectra Pro-750i, Acton Research Corporation, Acton, MA, USA), a 100-W bromine-tungsten lamp, and a lock-in amplifier (SR830 DSP, Stanford Research Systems, Sunnyvale, CA, USA) in comparison with reference Si and Ge cells [9]. In this work, the image was recorded at equal exposure time, and all the experiments were done in a dark room at room temperature.

### TJ solar cells

**Figure 2:**
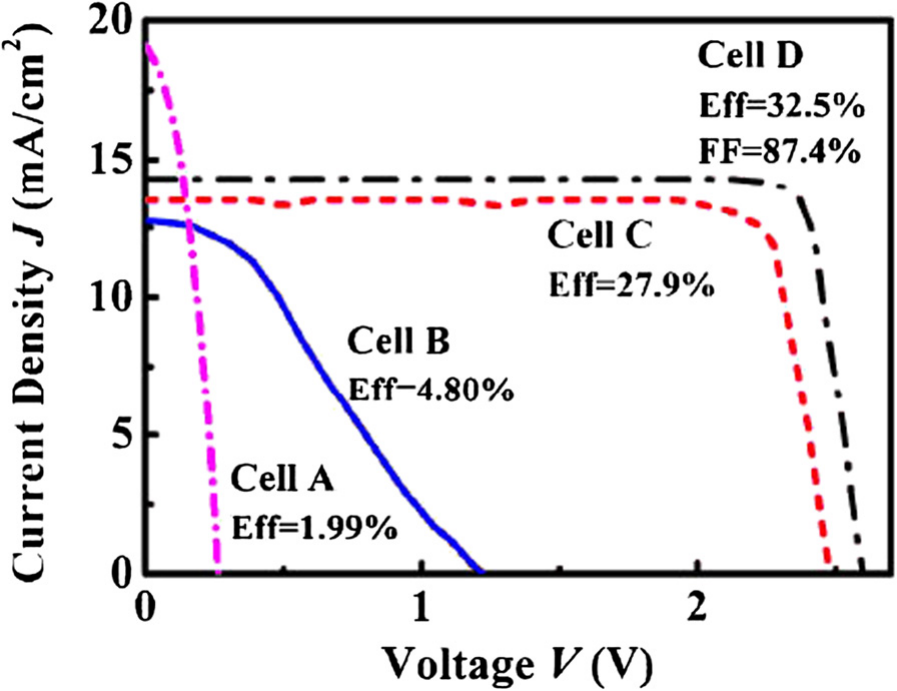
***I-V*** **characteristics with FF and efficiency of TJ solar cell samples.**

In this study, the TJ GaInP/GaInAs/Ge solar cells were grown by metal-organic chemical vapor deposition (MOCVD) with an active area of 5.6 × 5.9 mm^2^. To inspect instrument performance, four samples (labeled as A, B, C, and D) with different conversion efficiencies were characterized. Figure 2 shows their current-voltage (*I*-*V*) curves measured under AM1.5G illumination. The corresponding conversion efficiencies are marked in the figure. Cell A has the lowest conversion efficiency, and its open-circuit voltage is only 0.263 V, approximately equal to the open-circuit voltage of the Ge bottom cell. Cell B has a better performance than cell A, but its electrical parameters are still low. The steep *I*-*V* curves of cell A and cell B suggest the possibility of electric leakage. Among these cells, cells C and D are relatively normal, and the latter has the greatest conversion efficiency with the open-circuit voltage (*V*_OC_) and the short-circuit current density (*J*_SC_) of 2.60 V and 14.3 mA/cm^2^, respectively.

## Results and discussion

### Photographic diagnosis

**Figure 3:**
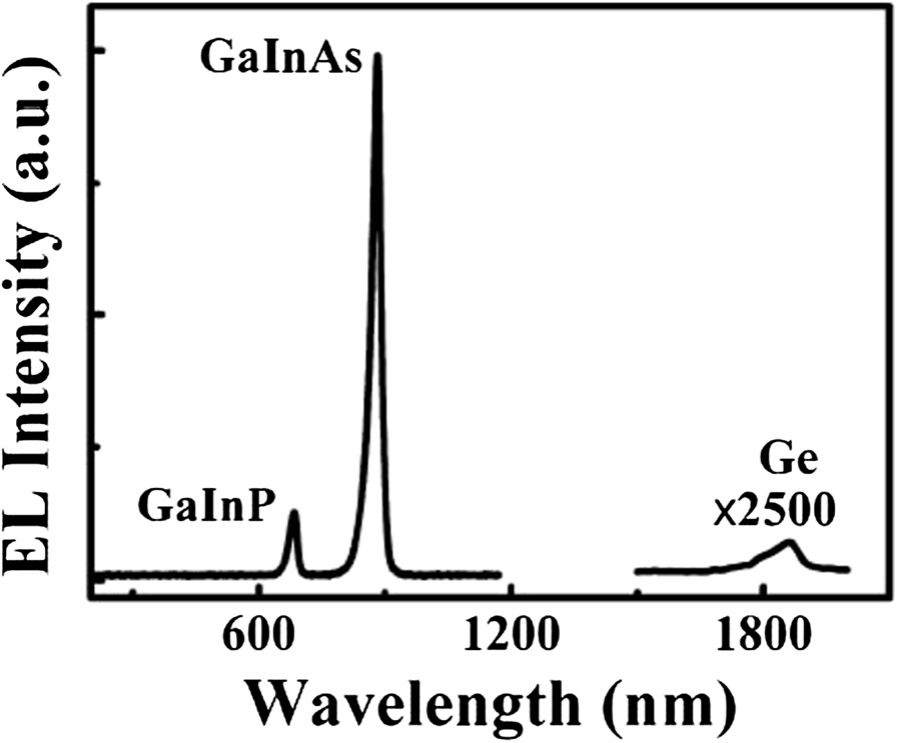
**Spectrally resolved EL spectrum of the TJ solar cell.**

To explore the factors affecting conversion efficiency, we first used the EL imaging method to detect the defects of the above samples. Figure 3 shows the spectrally resolved EL spectrum of cell D (similar to that of other cells). There are two strong peaks at 683 and 881 nm and one weak peak around 1,862 nm, which corresponds to the band edge emissions of the GaInP top junction, GaInAs middle junction, and Ge bottom subcell, respectively. Based on the position of emission peaks, visible vidicon was used to capture the photon emission from the GaInP top cell, while infrared vidicon together with an 800-nm IR filter was used to selectively receive signals from the GaInAs middle cell. It is noted that the EL peak of the Ge cell has been beyond the measuring range of the infrared vidicon. Hence, only the topmost two junctions were taken into account in this work, and such processing would not affect our later discussions. This is due to the fact that the photocurrent from the bottom cell is almost twice higher than that of the remaining subcells while its voltage contribution is small [10]. Moreover, the Ge bottom cell is usually formed by diffusion process rather than epitaxy growth, indicating a high material quality [11,12].

**Figure 4:**
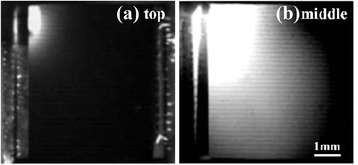
**EL images of GaInP (a) and GaInAs (b) subcells of cell A under a forward bias of 2.0 V.**

Figure 4 shows the EL images of cell A under a forward bias of 2.0 V. As shown in Figure 4a, almost no luminescence is observed for the GaInP top cell except at the upper left corner where the local intensity is very strong. It is noted that an extremely bright emission in the same position was found for the GaInAs middle cell in Figure 4b. Considering the injected current of 320 mA, it is believed that this solar cell has been badly impaired due to electric leakage, which agrees well with the above prediction. The degradation of the *I*-*V* parameters was mainly caused by a low shunt resistance and the increased current density in the upper left part. As for the top cell, the high current density spreading to the lower junction overcompensates the leakage defect, which leads to a dark EL image over the cell.

**Figure 5:**
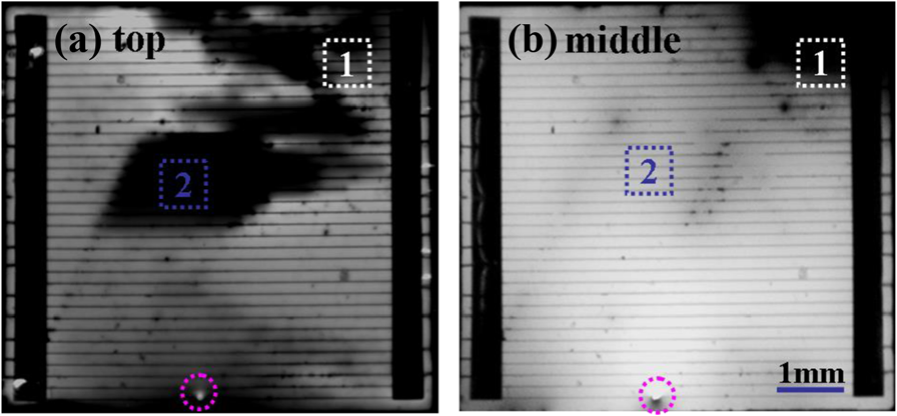
**EL images of top (a) and middle (b) subcells of cell B under 2.5 V forward bias.**

Figure 5 shows the EL images of cell B. Clearly, area ‘1’ in the top cell is brighter than that in the middle subcell due to the intrinsic defects of the GaInAs subcell. The existence of material defect makes this region become a recombination center, resulting in a weak emission. Meanwhile, area ‘2’ in the top cell is darker than that in the middle cell, and moreover, the shadow edge is parallel to the electrode, which means that the damage might originate from the fabricated electrode. When under EL, forward bias is applied on the wider bus; thus, the injected current flows from the wider bus through to the fingers while spreading laterally and vertically to the upper and lower junctions. Therefore, if there is any mechanical damage from the front electrode, the EL emission will be affected immediately and visible from the EL image of the top junction. As shown in Figure 5a, the relatively severe electrode damage has made the GaInP subcell large-scale nonluminous. Besides, we note that there is a bright spot (purple circle) in both subcells. This behavior generally indicates the occurrence of leakage at the point, which will greatly deteriorate the fill factor and photo-electric conversion efficiency.

**Figure 6:**
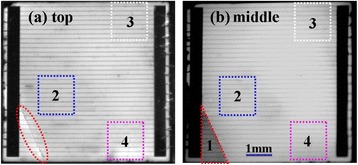
**EL images of GaInP (a) and GaInAs (b) subcells of cell C.**

**Figure 7:**
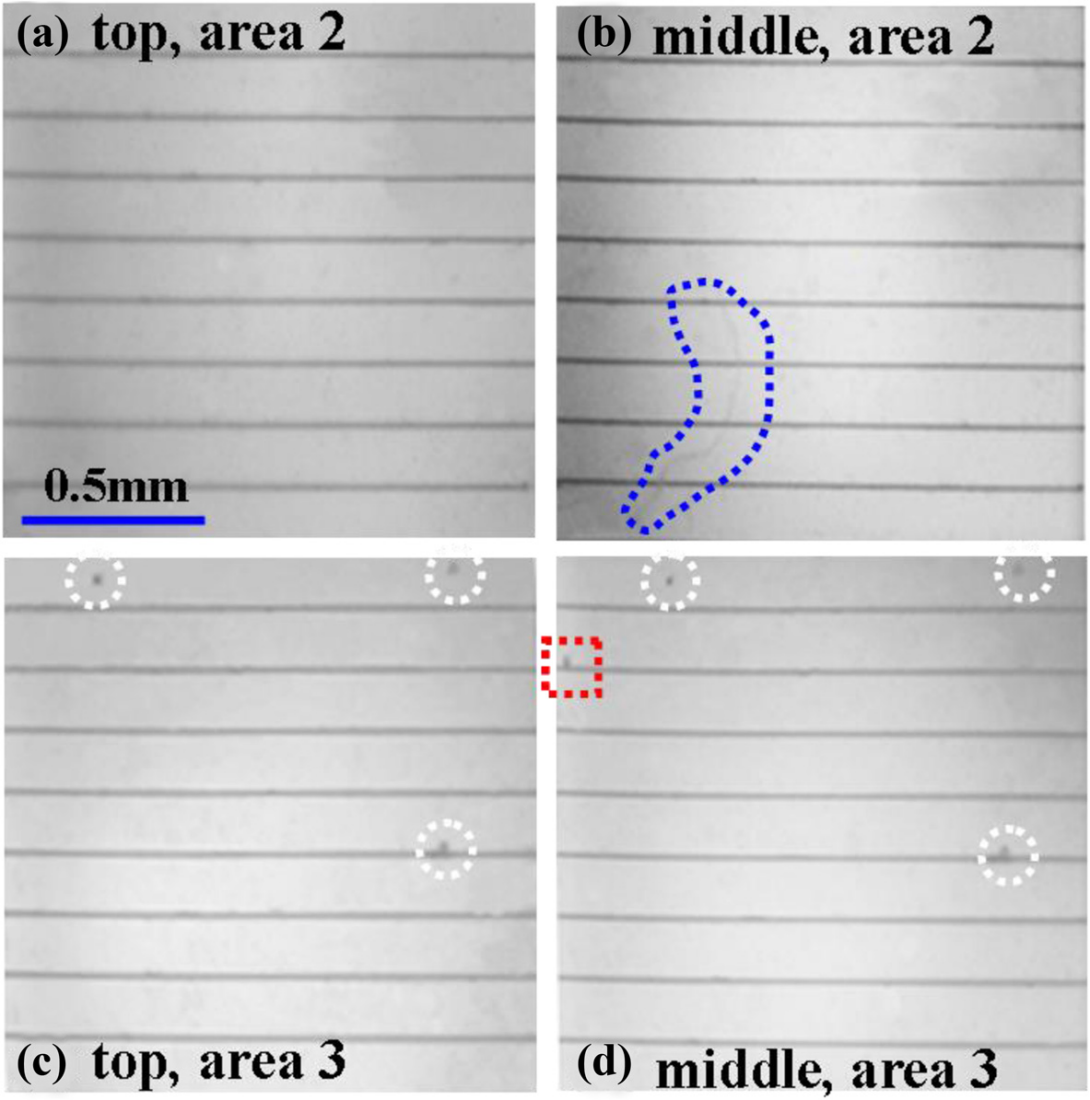
**Magnified EL images of GaInP and GaInAs subcells of cell C in different areas. (a, b)** Area 2 in Figure 6. **(c, d)** Area 3 in Figure 6.

Figure 6 shows the EL images of cell C. Compared with the above two samples, this cell has relatively bright and homogeneous images. There is only an obvious dark area at the lower left corner (area ‘1’) in Figure 6b, which originated from the micro crack (red dashed oval) of the GaInP subcell. To further study other defects, we examined cell C in the magnification lens. Figure 7 shows the magnified EL images of areas ‘2’ and ‘3’ in Figure 6. It is noted that there is a long winding line at the lower left corner (blue dash) in Figure 7b, whereas it is invisible in the GaInP subcell (Figure 7a). The line runs at random angles to the electric grid, and no gray difference appears on both sides. These phenomena indicate that the defect is a hidden crack. This inside crack is small so that cell C can still be used normally, but its long-term reliability will be affected [13,14]. As for area ‘3’, there are several dark spots marked by a circle on both subcells, which might be attributed to defects penetrating through all three subcells, such as threading dislocations. In addition, one dark spot marked by a square only exists in the middle subcell, indicating a defect located in the GaInAs subcell.

**Figure 8:**
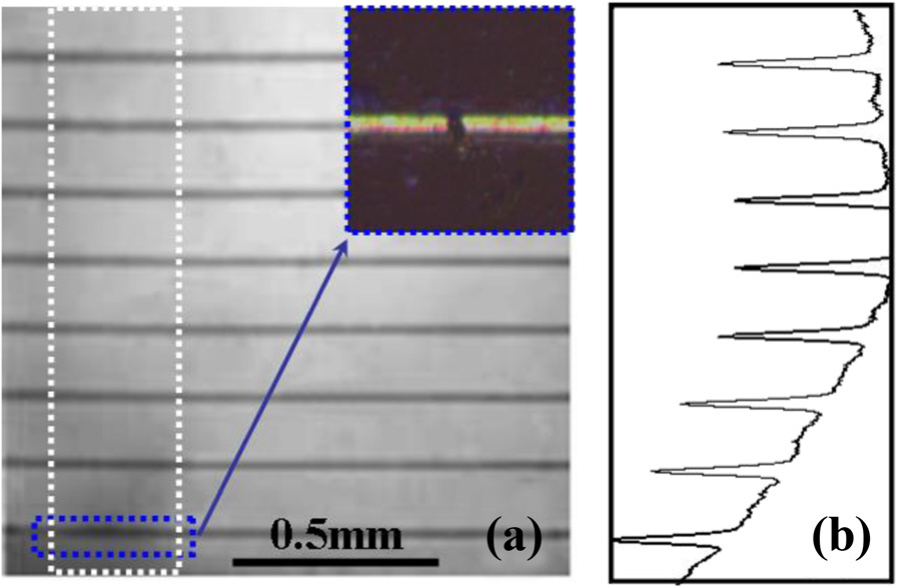
**Broken fingers detection of cell C. (a)** Magnified EL image of the GaInP subcell, area 4 in Figure 6. **(b)** Gray-scale curve for the vertical profile (white dashed square).

The EL imaging technique is also capable of detecting broken fingers [15]. Figure 8 illustrates the magnified EL image of area ‘4’ in Figure 6. The electrode grid labeled by a blue dashed square is darker than the others, indicating a broken line. The inset shows the magnified optical photograph of the selected finger and clearly exposes the broken part of the finger. Additionally, Figure 8b shows the gray-scale curve for the vertical profile (white dashed square) based on the data extracted from the image. It can be seen that the potential gradually decreases in the vicinity, which means that the broken finger affects not only the area beneath it but also the nearby region. This kind of defect will increase series resistance of a finished cell and correspondingly reduce conversion efficiency [16].

**Figure 9:**
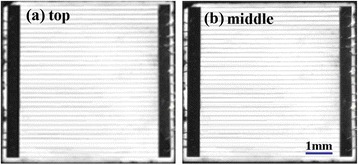
**EL images of GaInP (a) and GaInAs (b) subcells of cell D.**

Figure 9 presents the EL image of cell D. The EL image shows little spatial variations or inhomogeneities, indicating a better crystal quality and device structure. This result supports its high conversion efficiency.

### Quantum efficiency measurement

**Figure 10:**
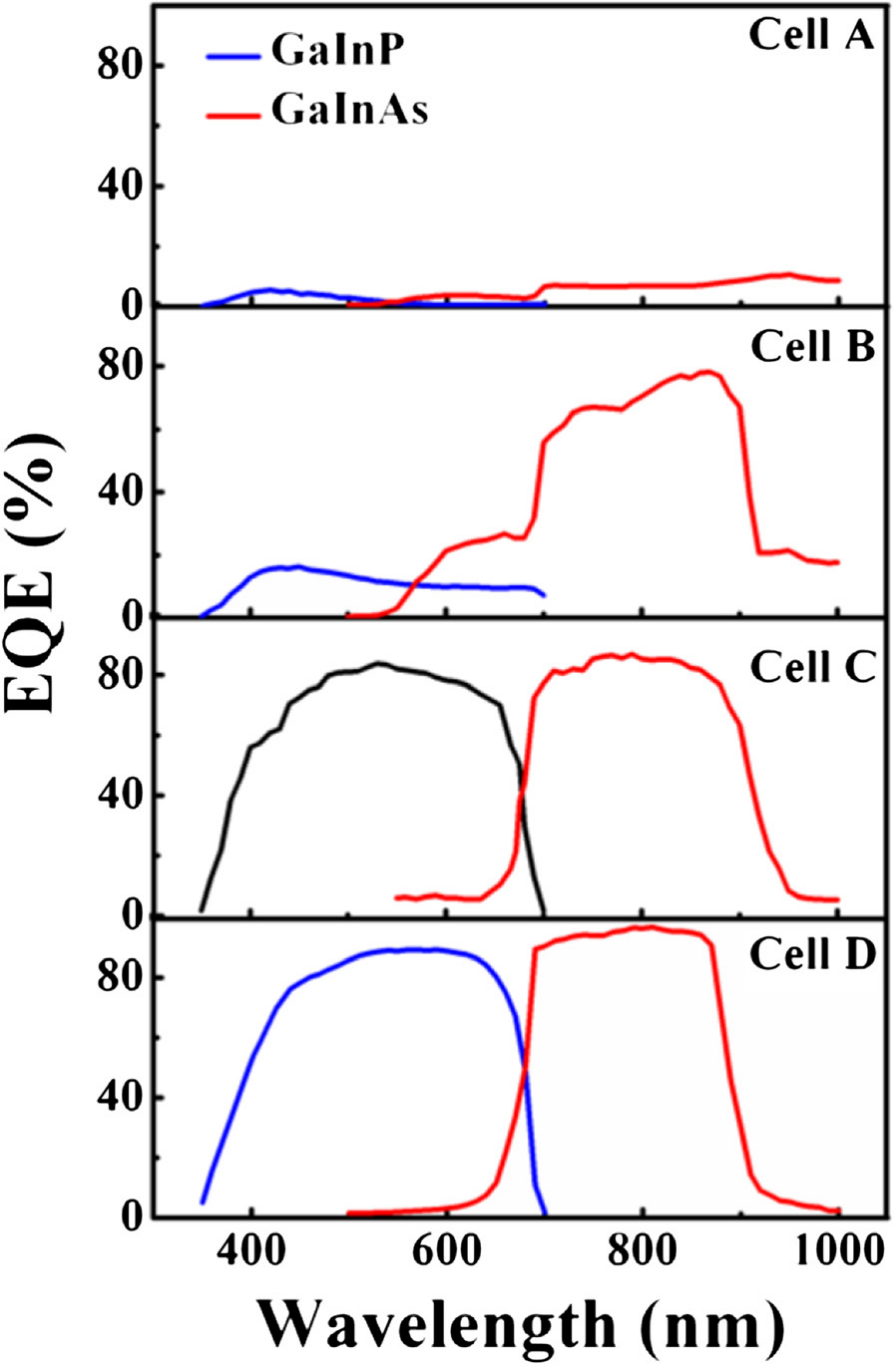
**EQE curves for different cell samples.**

It is known that the conversion efficiency of solar cells significantly depends on EQE, which can be affected by different kinds of defects in various degrees. To explore their relationship, we conducted EQE measurements for all cells with the results in Figure 10. As shown in Figure 10a, the quantum efficiencies for both junctions of cell A are very low, which are attributed to its serious electric leakage observed in Figure 4. During the EQE test, if there is a severe short-circuit phenomenon in a certain subcell, we could not obtain its EQE, even when a bias voltage was applied on the cell [17]. As for cell B, the EQE of the GaInP top cell is also far below the normal value, which is consistent with the large-scale nonluminous phenomenon in Figure 5a. Although the EQE of the GaInAs subcell is significantly higher than that of the GaInP subcell, the maximal value is still less than 80%, which might be explained by the previous EL images showing certain defects. Compared with cells A and B, cells C and D show better performances. The average EQE of cells C and D is about 80% and 90%, respectively. A maximal value of 96.7% was obtained in cell D at 810 nm. The slightly low EQE demonstrates that point defects or structural damages in a small area still play an unneglectable impact on conversion efficiency.

### Reciprocity relation between electroluminescence and quantum efficiency

Based on the analysis above, the EL image brightness significantly depends on the EQE. Here, we further theoretically explore their relationship. The basic theoretical ingredient for our analysis is the spectral reciprocity theorem [18]. At the coordinate *X* = (*x*, *y*) on the surface of the solar cell, the EL intensity *ϕ*_el_ can be given by [19-27]

1 $$ {\phi}_{\mathrm{e}\mathrm{l}}\left(E,X\right)={Q}_{\mathrm{e}}\left(E,X\right){\phi}_{\mathrm{bb}}(E)\left[ \exp \left(\frac{qV(X)}{kT}\right)-1\right] $$

where *Q*_e_(*X*) and *ϕ*_bb_ are the local external quantum efficiency and the black body photon flux with respect to the photon energy *E* of the EL peak of the subcell, respectively, *V*(*X*) is the local junction voltage, and *kT*/*q* is the thermal voltage.

Using the Boltzmann approximation for *ϕ*_bb_ as [22,26]

2 $$ {\phi}_{\mathrm{bb}}(E)=\frac{2\pi {E}^2/\left({h}^3{c}^2\right)}{ \exp \left(E/kT\right)-1}\approx \frac{2\pi {E}^2}{h^3{c}^2} \exp \left(\frac{-E}{kT}\right) $$

Equation 1 can be reformulated to

3 $$ {\phi}_{\mathrm{e}\mathrm{l}}\left(E,X\right)\approx \frac{2\pi }{h^3{c}^2}{Q}_{\mathrm{e}}\left(E,X\right){E}^2 \exp \left(\frac{-E}{kT}\right) \exp \left(\frac{qV(X)}{kT}\right) $$

where *h* and *c* are Planck's constant and vacuum speed of light, respectively.

In the experiment, the EL emission is first focused through a set of optical elements and then recorded by a charge-coupled device with a limited pixel (640 × 480). The transmission and conversion losses will result in the difference between the image brightness and the actual EL emission. The actual number of counts acquired by the vidicon can be given as [28]

4 $$ {\phi}_{\mathrm{ccd}}={\phi}_{\mathrm{el}}\times {C}_{\mathrm{img}}\left(\lambda \right)+{C}_{\mathrm{off}} $$

where *C*_img_(*λ*) is the imaging constant, which is related to multiple factors, such as vidicon quantum efficiency, vidicon conversion factor [counts/electron], maximum transmittance at the center of all optical elements, and geometry factor for the fraction of EL radiation that enters the optical elements. Since the vidicon signal loses spectral information, we assumed that the value is a constant one here. *C*_off_ represents the actual zero pixel value, which can be obtained by acquiring an absolutely dark image. Based on the above equations, *ϕ*_ccd_ of a raw EL image obtained directly from the vidicon can be expressed as

5 $$ {\phi}_{\mathrm{ccd}}\left(E,X\right)\propto {Q}_{\mathrm{e}}\left(E,X\right){\phi}_{\mathrm{bb}}(E){C}_{\mathrm{img}} $$

Here, we suppose that the local voltage *V*(*X*) is a constant [29]. It is clear that the intensity of the EL image is proportional to the EQE. Additionally, the short-circuit current density *J*_SC_ of the solar cell can be calculated from the EQE by

6 $$ {J}_{\mathrm{SC}}=\frac{q}{hc}{\displaystyle \int}\lambda {\phi}_{\mathrm{sun}}\left(\lambda \right){Q}_{\mathrm{e}}\left(\lambda \right)d\lambda $$

where *λ* is the wavelength, *h* and *c* have the same meaning as above, and *ϕ*_sun_ (*λ*) is the solar radiation spectrum.

Ideally, the EQE in the response range of the solar cell just fluctuates around a certain value, as shown in Figure 10, which is written as $\overline{Q_{\mathrm{e}}}$ here. Hence, we rewrite the Equation 6 as follows:

7 $$ {J}_{\mathrm{SC}}\approx \frac{q}{hc}\overline{Q_{\mathrm{e}}}{\displaystyle \int}\lambda {\phi}_{\mathrm{sun}}\left(\lambda \right)d\lambda $$

For a certain material, the integral term is a constant. As a result, Equation 5 can be deduced as

8 $$ {\phi}_{\mathrm{ccd}}\left(E,X\right)\propto {J}_{\mathrm{SC}}{\phi}_{\mathrm{bb}}(E){C}_{\mathrm{img}} $$

In order to verify Equation 8, we used an image processing software to obtain the pixel gray values of EL images. Then, we calculated the mean gray value $\left({\overline{\phi}}_{\mathrm{ccd}}\right)$ as follows:

9 $$ {\overline{\phi}}_{\mathrm{ccd}}=\frac{1}{S_{\mathrm{aa}}}{\displaystyle \underset{s_{\mathrm{aa}}}{\int }}{\phi}_{\mathrm{ccd}}\left(E,X\right)\cdot dS $$

where *S*_aa_ is the active surface area of the solar cell.

**Figure 11:**
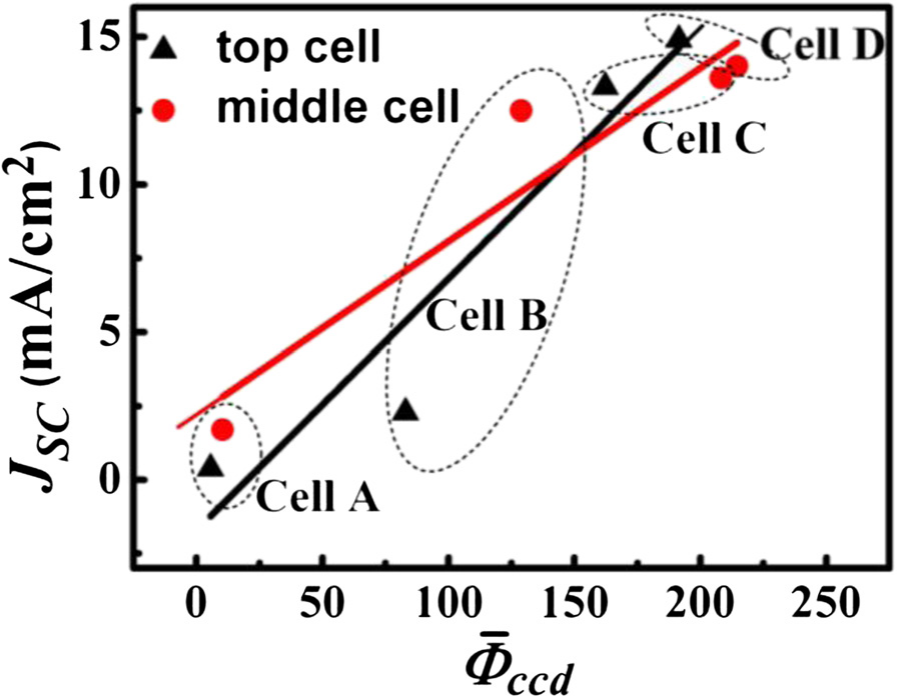
**The dependence of mean gray value of EL images on** ***J***_**SC**_ **.**

In the experiment, automatic gain adjustment in the image acquisition software is disabled and the injected current was kept at 20 mA. Figure 11 illustrates the dependence of the photocurrent density from each junction on the averaged EL intensity. It can be seen that the current density is almost proportional to EL intensity for both the subcells, which agrees well with Equation 8. This behavior means that we can obtain the current density by EL microscopy technology with the calibrated parameters, and it will provide a simple and effective method for the characterization of the current matching condition of MJ solar cells.

## Conclusions

In conclusion, the EL imaging technique for MJ solar cells was established by combining EL imaging with EL spectroscopy. By comparing the images taken from each subcell, different defects or damages can be definitely identified. The EL imaging system was proved to be a powerful diagnostic tool for investigating not only the material properties but also process-induced deficiencies in MJ solar cells. The EQE results confirmed different types of defects or damages impacting cell performance in various degrees, and the electric leakage mostly degraded the EQE. Moreover, the relationships between the gray value of the EL image and EQE or *J*_SC_ were deduced and discussed. The results showed that the gray value was almost proportional to EQE or *J*_SC_. It is believed that this method will provide a simple and effective method with the calibrated parameters for evaluating the current matching status of MJ solar cells.
